# Asymmetric posterior reversible encephalopathy syndrome in patient with hyperplastic anterior choroidal artery

**DOI:** 10.1007/s10194-010-0284-2

**Published:** 2011-01-05

**Authors:** Andrea Romano, Pugliese Silvia, Pierallini Alberto, Francesca Tavanti, Giuliano Sette, Sara La Starza, Luigi Maria Fantozzi, Alessandro Bozzao

**Affiliations:** 1 Department of Neuroradiology, S. Andrea Hospital, University “La Sapienza”, Via di Grottarossa 1035, 00189 Rome, Italy; 2 IRCSS San Raffaele Pisana, Rome, Italy; 3 Department of Neurology, S. Andrea Hospital, University “La Sapienza”, Rome, Italy

**Keywords:** Anterior choroidal artery, PRES, Vasogenic edema, Hypertension, Headache

## Abstract

We describe a case of asymmetric PRES due to the presence of hyperplastic anterior choroidal artery (AChA) in a man affected by sever hypertension. Posterior reversible encephalopathy syndrome (PRES) has become synonymous with a unique pattern of brain vasogenic edema and predominates in the parietal and occipital regions, accompanied by clinical neurological alterations. Sever hypertension is a risk factor that exceeds the limits of brain autoregulation, leading to breakthrough brain edema. In our knowledge this is the first case reported in literature, in which a similar vascular abnormality is linked to a PRES syndrome.

## Introduction

Posterior reversible encephalopathy syndrome (PRES) relates to a transient pattern of brain edema mostly involving the parietal and occipital regions. Severe hypertension may lead to PRES when overcoming brain autoregulation with subsequent breakthrough brain edema. MR imaging demonstrates reversible and symmetric focal hemispheric edema. We describe a case of asymmetric PRES associated with the presence of a hyperplastic anterior choroidal artery (AChA) in a man affected by severe hypertension.

## Case report

A 58-year man, with a history of mild hypertension, was referred to the emergency room for a sudden onset of visual disturbance, difficulty in speech, headache and mental confusion. Clinical examination revealed increased blood pressure (220 mmHg), mild anemia (HGB, 10.5 g/dl) and hyper-cholesterolemia (250 mg/dl) on routine blood work.

Neurologic examination revealed left homonymous lateral hemianopia, mild difficulty in searching words, mild pronation and downward drift of the right arm.

Electro-encephalography was positive for left temporo-occipital alterations.

CT revealed bilateral cortical–subcortical patchy hypodense foci localized in the parietal-occipital lobes, asymmetric for left predominance without enhancement after i.v. injection. Multiple hypodense confluent areas localized in the supratentorial white matter as a result of chronic ischemic cerebral disease were evident as well.

**Fig. 1:**
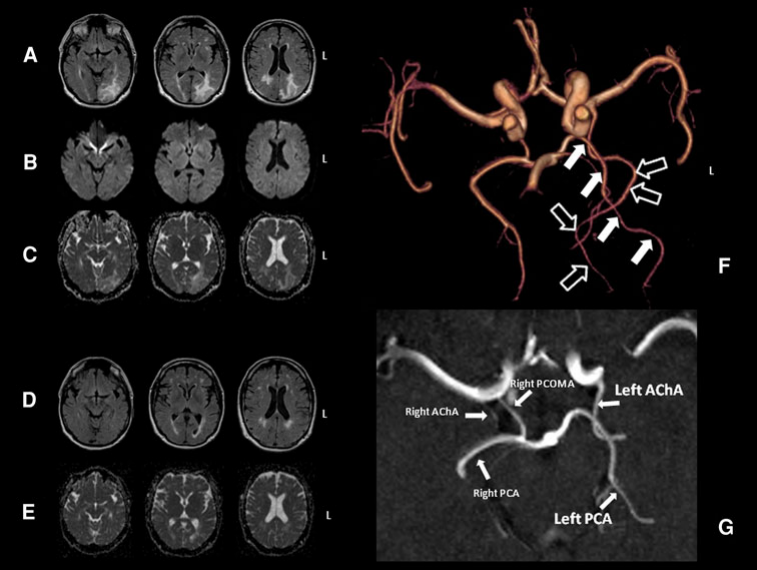
The first MRI (**a**–**c**) showed cortical–subcortical patchy hyperintense areas on T2 FLAIR-weighted images (**a**) localized in the parietal–occipital lobes with asymmetric representation for left predominance. No signal alteration was seen in DWI images (**b**) in presence of high signal on ADC maps indicating vasogenic edema (**c**). MRI exam after 3 months demonstrated complete resolution of the hyperintense areas on FLAIR-weighted (**d**) images and ADC maps (**e**). The MR-angiography of intracranic circle, performed with the 3D-TOF technique (**f**, **g**), showed the presence of left hyperplastic AChA (*white arrows*, tridimensional images **f**) supplying partially the distribution of the ipsilateral posterior cerebral artery (*PCA*) (*arrows*, tridimensional images **f**). In **g** the same anatomical abnormalities is represented by MIP reconstruction

MRI, performed immediately after CT, showed the presence of asymmetric cortico-subcortical patchy hyperintense areas on FLAIR images mostly localized in the left parieto-occipital lobe (Fig. 1a). No signal alteration was seen in DWI images while ADC maps showed high signal indicating vasogenic edema (Fig. 1b, c). No pathological enhancement was seen after contrast administration.

MR-angiography, performed with the 3D-TOF technique, showed the presence of left hyperplastic AChA partially supplying the distribution of the ipsilateral posterior cerebral artery (PCA) (Fig. 1f–h).

Because of the suspicion of acute hypertensive encephalopathy, he was treated with mannitol (100 mL 6/day), potassium canrenoate (50 mg 1cp/day), furosemid (25 mg 1cp/day).

Seven days after admission, symptoms resolved, blood pressure was 140/90 mmHg.

Eleven days after admission, the patient was discharged with complete resolution of the symptoms, stable reduction of the blood pressure (115/70 mmHg) and a therapy composed by potassium canrenoate (50 mg 1cp/day), ramipril (10 mg 1cp/day), doxazosin mesylate (2 mg 1 cp/day), amlodipine besylate(10 mg 1 cp/day) and acetylsalicylic acid (100 mg 1cp/day).

MRI follow-up performed 3 months later demonstrated complete resolution of the signal alterations (Fig. 1d, e).

## Discussion

The AChA is a branch of the internal carotid artery running between the temporal lobe and the brain stem and reaching the choroid plexus in the temporal ventricular horn. Along its course, the AChA supplies several territories, in particular optic tract, posterior limb of internal capsule and pulvinar. Several anomalies in course and caliber have been described. In particular, enlargement of the AChA has been reported with cerebral angiography in many pathological cases [1]. In up to 1% of subjects, the AChA is hyperplastic and supplies all, or a portion, of the PCA territory. Takahashi et al. [2] described 25 hyperplastic arteries supplying part of the territory of distribution of the PCA. The hyperplastic arteries were further classified into four subtypes according to the distribution area and course of the vessel. In type 3 an anomalous artery supplies the parieto-occipital and calcarine arteries [3], as observed in our patient. This is a result of anastomotic channels development, hemodynamically favored, between AChA and PCA or PCOMA that determined variable degrees of hypoplasia of the PCA [3].

In our patient the hyperplastic AChA supplied the territory affected by T2 signal alteration, suggesting a potential role of the vascular anomaly in its genesis.

PRES is an acute rapidly evolving clinical condition characterized by headache, nausea and vomiting, abnormalities of visual perception, mental status abnormalities, seizure and focal neurological signs associated with transient radiological brain anomalies. Different conditions might be responsible (eclampsia, hypertensive encephalopathy, renal disease with hypertension, neurotoxicity of cyclosporine A or other immunosuppressive drugs and bone marrow transplantation) [4–7]. Other rare pathologic conditions, such as intracranial hypotension, are recently discussed [8, 9].

The presence of hypertension and the breakthrough of cerebral autoregulation are retained as the most common causes of PRES.

As mentioned radiological findings are usually reversible [5, 7]. MR diffusion-weighted imaging usually confirms the presence of vasogenic edema with high values of ADC.

In our patient, many findings were compatible with PRES. Clinical onset and symptoms (headache with nausea, visual disorders, altered mental status and seizure) were evident in acute phase and completely resolved at discharge. In our patient, the choice of using anti-hypertensive care was due to the difficulty of a fast reduction of blood pressure, avoiding other complications [10].

Lesion distribution in MRI was characteristic of PRES as well, although clearly asymmetric. Common involvement of atypical brain location other than parietal or occipital regions is known [11]. The authors in this study assessed that partial or asymmetric PRES was most commonly recognized in patients who have had organ transplantation and eclampsia with severe hypertension or normal blood pressure. Severe hypertension, renal failure, immunosuppression could be also associated with asymmetric PRES [12]. Variable expression of the PRES patterns could be related to differences in arterial anatomy, as demonstrated in our case, preexisting vascular disease or regional hemispheric involvement in a clinical toxic process [11].

Acute severe hypertension with transient loss of autoregulation system (sympathetic innervations) and subsequent vasogenic edema [7, 11] seems the cause of PRES in our patient. Posterior distribution of brain alterations has been attributed to the sympathetic innervations of the cerebral vessels. This distribution has an antero-posterior gradient with a relatively reduced innervation of the posterior cerebral arterial circulation [13], and therefore, during acute elevation of blood pressure, posterior brain regions may be particularly susceptible to breakthrough of autoregulation. The presence of a “double” vascular district (AChA and PCA) in the same vascular territory during acute hypertensive attack might be the cause of “blood hyper-influx syndrome” leading to asymmetric vasogenic edema.

Although the presence of hyperplastic AChA is well known [2], to our knowledge this is the first case reported in literature in which such a vascular abnormality was linked to PRES syndrome.
